# COVID-19-related headache and sinonasal inflammation: A longitudinal study analysing the role of acute rhinosinusitis and ICHD-3 classification difficulties in SARS-CoV-2 infection

**DOI:** 10.1177/03331024211040753

**Published:** 2021-09-20

**Authors:** Marcin Straburzyński, Magdalena Nowaczewska, Sławomir Budrewicz, Marta Waliszewska-Prosół

**Affiliations:** 1 General Practice, Orzyny, Poland; 2 Headache Clinic – Terapia Neurologiczna ‘Samodzielni’, Warsaw, Poland; 3 Department of Otolaryngology, Head and Neck Surgery and Laryngological Oncology, Ludwik Rydygier Collegium Medicum in Bydgoszcz, Nicolaus Copernicus University, Bydgoszcz, Poland; 4 Department of Neurology, 49550 Wroclaw Medical University, Wroclaw Medical University, Wroclaw, Poland

**Keywords:** Sinusitis, facial pain, ICOP

## Abstract

**Background:**

The genesis of headache in coronavirus disease 2019 (COVID-19) is currently unclear and the multitude of disease symptoms often further hinders locating the source of pain. Interestingly, many subjects with COVID-19 have symptoms of acute rhinosinusitis. The relation between nasal symptoms and headache in SARS-CoV-2 infection remains unknown.

**Methods:**

This bi-center longitudinal study evaluated symptoms in consecutive COVID-19 patients in the participating practices. The first assessment was performed during the initial consultation after infection confirmation. That was followed up by a second consultation after a median 9 days.

**Results:**

130 patients were included in the study (80 women, 50 men; mean age 46.9 years). Headache was highly prevalent at the first visit (72%) and significantly associated with acute rhinosinusitis symptoms. The odds ratio for headache in subjects with rhinosinusitis was 3.5. Headache could be attributed to systemic viral infection in 96% and to acute rhinosinusitis in 51% of cases according to 3rd edition of the International Classification of Headache Disorders. Criterium C.3 (exacerbation of headache by pressure applied over paranasal sinuses) and C.4 (ipsilaterality of headache and sinusitis) had low sensitivity in headache attributed to acute rhinosinusitis.

**Conclusions:**

Nasal inflammation is associated with headache in COVID-19, although the pain mechanism lies probably in a systemic reaction to the virus. 3rd edition of the International Classification of Headache Disorders criteria for headache attributed to acute rhinosinusitis need adjusting to the current understanding of acute sinonasal infection.

## Introduction

Headache is one of the most prevalent symptoms of coronavirus disease 2019 (COVID-19). This observation has been confirmed by studies prospectively collecting data in structured questionnaires (1–3). However, little is known about the pathogenesis of this symptom. Two of the most popular hypotheses attribute headache to either a viral systemic infection (chapter 9.2.2.1 according to the 3rd edition of International Classification of Headache Disorders (ICHD-3) or viral meningitis or encephalitis (9.1.2.1/2) (4–6). The first of these diagnoses seems especially prevalent and manifests with a plethora of systemic and local symptoms, including fever, malaise, cough, dyspnoea, diarrhoea, nasal discharge and smell and taste impairment. These symptoms resemble complaints observed in other viral infections, such as influenza or common cold. The latter has been predominantly caused by rhinoviruses and, even before the current pandemic, coronaviruses. In these viral infections the majority of patients have symptoms of an acute mucosal inflammation of the nose and paranasal sinuses (7). This regional inflammation often called ‘common cold’, is presently named ‘acute rhinosinusitis’ (ARS). Headache caused by ARS has its own criteria in ICHD-3 (11.5.1) (6). The most recent definition of ARS was published in the 2020 European Position Paper on Rhinosinusitis and Nasal Polyps (EPOS) (7). According to this document, ARS can be clinically diagnosed when symptoms presented in Table 1 occur. Since the symptoms of COVID-19 allow in many cases to diagnose ARS, the purpose of this study was to examine the hypothesis that headache in COVID-19 can be attributed to ARS. Moreover, findings from this study will help to analyse the value of current ICHD-3 diagnostic criteria of headache attributed to ARS, one of the most prevalent secondary headaches.

**Table 1.** Definition of acute rhinosinustitis (ARS) from European Position Paper on Rhinosinusitis and Nasal Polyps (7).

ARS in adults	ARS in children
sudden onset of two or more symptoms, one of which should be either nasal blockage/obstruction/congestion or nasal discharge (anterior/posterior nasal drip):• ± facial pain/pressure,• ± reduction or loss of smell for <12 weeks	sudden onset of two or more of the symptoms:• nasal blockage/obstruction/congestion• or discoloured nasal discharge• or cough (daytime and night-time) for <12 weeks

## Materials and methods

This study has a bi-center longitudinal design and was conducted in a rural primary-care practice and a large university hospital. The target group included consecutive patients with symptomatic SARS-CoV-2 infection confirmed by either polymerase chain-reaction or antigen test (Panbio™ COVID-19 Ag Rapid Test Device). In the general practice, the participants were recruited from all subjects who reported for consultation. In the hospital, the group included all new-onset COVID-19 cases consulted by the investigator (MWP) within the emergency and outpatient department. The subjects had to be above 6 years old and give informed consent to participate in the study. The research was conducted according to the principles of the Declaration of Helsinki and the study protocol was submitted for the Ethics Committee of the Warmia and Mazury Medical Chamber in Olsztyn (no formal approval for observational study was required).

Exclusion criteria included: chronic rhinosinusitis, allergic and non-allergic rhinitis, recurrent acute rhinosinusitis, cystic fibrosis, primary ciliary dyskinesia, history of intranasal or systemic steroid use in the last 3 months, history of nasal surgery or trauma in the last 12 months, history of radiation therapy within nasal cavity or paranasal sinuses, chronic hyposmia or anosmia, recurrent or chronic problems with breathing through the nose, cognitive and neurologic disorders preventing obtaining a reliable medical history.

All subjects included in the study were evaluated twice by a physician specialising in headache medicine, i.e. a general practitioner with special interest in headache (MS) or neurologist (MWP). Data was collected either in person during a medical appointment, or using telemedicine solutions (video chat or telephone). A structured questionnaire was used to ensure a unified data extraction. The first assessment was performed after obtaining a positive SARS-CoV-2 test result (2–6 days from the onset of symptoms). A second evaluation was performed 7–12 days after the first examination.

Data collected included prior diagnosis of headache disorder, information about pain characteristics (i.e. location, intensity, duration, quality, exacerbation by physical activity) and nasal symptoms (hyposmia/anosmia, nasal discharge quality, facial pressure), accompanying symptoms (i.e. photophobia, phonophobia, osmophobia, nausea, vomiting, cranial autonomic symptoms). If applicable, symptoms were evaluated for laterality. Facial pain or headache was registered as present, even if the subject was pain-free, but had taken analgesics for headache or facial pain up to 12 hours prior to assessment.

In case of teleconsultations, patients were instructed to moderately press areas over, under and medially of the orbital rim. This was done to confirm exacerbation of headache by pressure applied over the paranasal sinuses. Other COVID-19 symptoms (e.g. fever, cough, myalgia, dyspnoea) were noted and used for assessing the presence of a systemic viral infection. When a pre-existing primary headache occurred during COVID-19, it was classified as both primary and secondary headache.

All subjects were screened for disease progression; severe cases were identified and admitted to hospital. The screening process included assessment of dyspnoea and partial CURB-65 (confusion, respiratory rate, blood pressure, age ≥65) performed during each consultation. Patient risk of progression to severe COVID-19 was assessed at the inclusion. The risk factors were identified according to Polish recommendations based on patients age, comorbidities and medications (8). Subjects flagged as ‘high-risk’ were assessed in surgery or during a house visit by the investigator. Moreover, high-risk patients were screened by serial measurements of oxygen saturation using government-sponsored pulse oximeters home-delivered after the initial assessment. Data interpretation was based on ICHD-3 (6) and EPOS (Table 1) criteria. In this regard the symptoms were assessed separately at both visits. In other words, if a symptom was present only at one visit, it was regarded as present only at that visit. Causality was assessed according to ICHD-3 – when headache and other symptoms occurred together (present at first visit), and then remitted together (absent or decreased at second visit), they were deemed to fulfil ICHD-3 reasoning (6).

The statistical analysis was made in the statistical environment R (version 3.6.0) and SPSS software. The results of *p* < 0.05 indicate significant relationships between the variables. Variables expressed at the ordinal or nominal level were analysed using chi-square tests. In the case of the fourfold contingency tables, the continuity correction was used, while when the conditions for the chi-square test were not met, the Fisher exact test was used with expansion for tables larger than 2 × 2. Parametric tests (student's T test or ANOVA) or their non-parametric equivalents (Mann-Whitney U test or Kruskall-Walis test) were used to analyse quantitative variables divided into groups. The selection of tests was made on the basis of variable distribution verified with the Shapiro-Wilk test.

## Results

**Figure 1.**
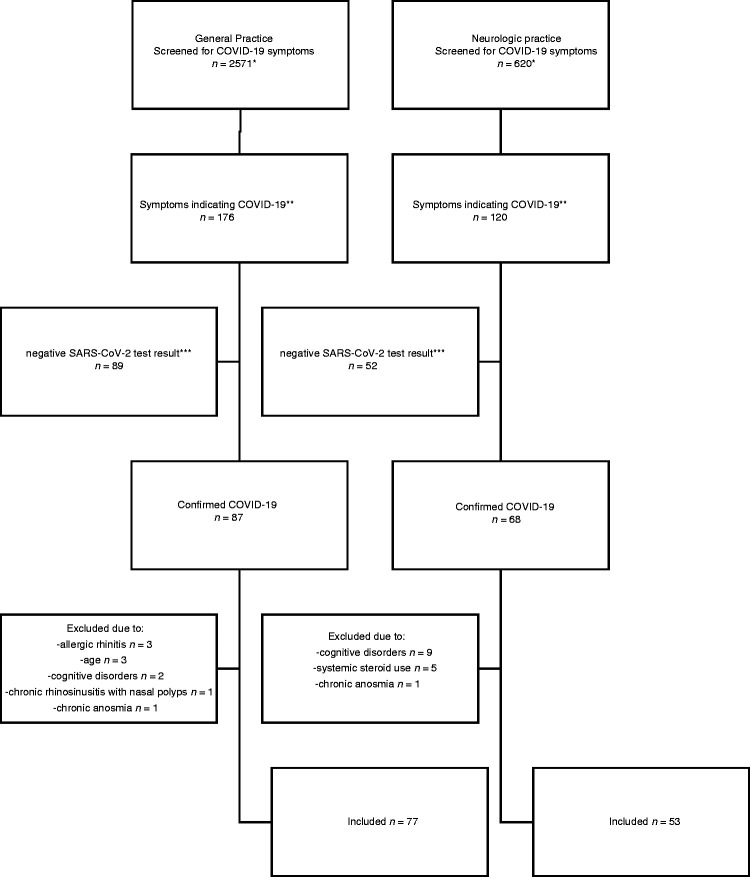
Diagram of patients’ selection process. *Screening was done at every appointment, the remaining numbers reflect patients. **New onset: cough, dyspnoea, fever, hypo-/anosmia, hypo-/aegusia, headache, facial pain, nasal discharge or congestion, sore throat, diarrhoea, myalgia, fatigue, rash, conjunctivitis. ***Two negative polymerase chain-reaction (PCR) test results or a negative antigen and a negative PCR test.

130 COVID-19 symptomatic patients (80 women, 50 men, aged 7–74 years, mean 41.6) were included in this study between 1 November 2020 and 19 March 2021 (Figure 1). None of the patients dropped out from the study due to lack of follow-up assessment. 59% of subjects were recruited from primary care and 41% from an outpatient neurology practice. Video- and teleconsultations consisted 48% of all appointments (91% second assessments). Table 2 presents the demographics and symptoms recorded during consultations. 22% of patients were diagnosed with migraine and 16% with tension-type headache (TTH) based on medical history and complaints preceding the onset of COVID-19. The first consultation was performed at median 3rd day from the beginning of COVID-19 symptoms. That was followed by a second consultation 9 (median) days later. Groups recruited in general and neurology practices were similar in regard to demographic data or time from disease onset to assessment (*p* > 0.05). However, ARS was more prevalent in the general practice (*p* = 0.027).

**Table 2.** Demographics and clinical characteristics of patients included in the study.

Demography
Age
7–74 (mean 41.16, SD 15.93, median 39)
Sex
Female *n =* 80 (61%)
Male *n* = 50 (38%)
Investigation sites
General practice *n =* 77 (59%)
Neurology practice *n =* 53 (40%)
Pre-existing headache disorder
None *n =* 76 (58.5%)
Migraine *n =* 28 (21.5%)
Tension-type headache *n =* 21 (16%)
Other *n =* 5 (3.8%)
COVID-19 course
Days from COVID-19 symptoms onset to the 1st assessment
1–6 (mean 2.86, SD 1.14, median 3)
Days from COVID-19 symptoms onset to the 2nd assessment
9–17 (mean 11.88, SD 1.54 median 12)
Headache and/or facial pain
at first assessment *n =* 93 (72%)
○ onset of headache with other COVID-19 symptoms *n =* 82 (89.1% of headache cases)
at second assessment *n =* 14 (11%)
Persistence of other upper respiratory tract symptoms at the 2nd assessment *n* = 43 (33%)
(more than one symptom per subject possible)
smell impairment *n* = 27 (21%)
nasal discharge *n* = 19 (15%)
facial pressure *n* = 3 (2%)
Severity assessment
high-risk subjects identified at first assessment *n =* 24 (18%)
○ headache subjects in that group *n* = 13 (54%)
hospitalised subjects *n* = 4 (3%)
○ headache subjects in that group *n* = 1 (25%)
Headache features
Pain location (more than one location per subject possible)
Forehead *n =* 55 (59%)
Diffuse *n =* 20 (22%)
Temporal *n =* 17 (18%)
Occipital *n =* 9 (10%)
Parietal *n =* 2 (2%)
—
Facial & forehead *n =* 59 (63%)
Facial pain without headache *n =* 4 (4%)
—
Unilateral headache or facial pain *n =* 6 (7%)
Pain intensity
Mild *n =* 5 (5%)
Moderate *n =* 38 (40%)
Severe *n =* 44 (47%)
Very severe *n =* 6 (7%)
Pain quality
pressing or tightening *n =* 70 (75%)
pulsating *n =* 23 (25%)
Accompanying symptoms
Smell impairment *n =* 55 (42%) including:
anosmia *n =* 28 (51%)
hyposmia *n =* 27 (49%)
Facial pressure *n =* 29 (22%)
Nasal discharge (anterior or posterior nasal drip) *n =* 52 (40.0%)
watery/mucoid *n =* 41 (79%)
discoloured *n =* 11 (21%)
—
bilateral *n =* 51 (98%)
unilateral *n =* 1 (2%)
Nasal congestion *n =* 39 (30%)
bilateral *n =* 38 (89%)
unilateral *n =* 4 (10%)
Headache and/or facial pain with non-nasal cranial autonomic symptoms *n =* 14 (15.1%)
conjunctival injection *n =* 9 (64%)
eyelid oedema *n =* 3 (21%)
lacrimation *n =* 2 (14%)
Autonomic symptoms (more than one symptom per subject possible)
nausea *n =* 26 (20%)
vomiting *n =* 11 (9%)
–
photophobia *n =* 18 (14%)
phonophobia *n =* 6 (5%)
osmophobia *n =* 1 (1%)
Headache and/or facial pain exacerbated by pressure applied over the paranasal sinuses *n =* 25 (27%)
ARS subjects in that group *n =* 13 (52%)
Other COVID-19 symptoms
Cough *n =* 50 (38%)
Fever *n =* 55 (42%)
Data interpretation
Pain phenotype
tension-type (including probable tension-type) *n =* 63 (68%)
migraine (including probable migraine) *n =* 27 (29%)
unclassified *n =* 3 (3%)
Clinical diagnosis of ARS *n =* 58 (45%)
Clinical diagnosis of acute bacterial rhinosinusitis *n =* 0 (0%)
Headache can be attributed to ARS (ICHD-3) *n =* 47 (51%)
Headache improvement or resolution in parallel with symptoms of systemic viral infection
n = 89 (96%)

SD – standard deviation, ARS – acute rhinosinusitis.

The patients were recommended only symptomatic treatment with paracetamol or ibuprofen. No subjects were prescribed systemic or inhaled corticosteroids. Of the included patients, 24 had higher risk of severe COVID-19 and 4 subjects (3%) were eventually hospitalised. These numbers disallowed a statistical search for associations between headache or ARS and patients status or oxygen saturation. Hospitalised patients were prescribed high flow oxygen (*n* = 4), remdesivir (*n* = 3), antibiotics (*n* = 3) and convalescents plasma (*n* = 3).

Headache was reported by 72% of patients at first consultation, and 11% at the second. The location of pain most often included the forehead (59%), with 94% patients reporting its bilateral distribution. Facial pain without headache was observed in 4% of participants. The majority of patients reported moderate to severe pain intensity, with pressing or tightening quality in 75% of cases. Headache and/or facial pain was accompanied by nausea in 20% of subjects. 14% of patients reported photophobia, 5% phonophobia, while osmophobia was noted by one participant. The investigators classified headache to have migraine phenotype in 29% and tension-type in 68% of cases. No association between pre-existing headache disorder and COVID-19-related headache phenotype was found (*p* = 0.11). Pain was exacerbated by pressure applied over the paranasal sinuses in 27% patients. Odds ratio for this symptom in ARS was 1.44 (confidence interval (CI) = 0.60–3.46, *p* = 0.55). Assessment of this symptom during teleconsultations did not differ significantly from physical examination by a practitioner (*p* = 0.85). Due to a limited number of subjects with pre-exiting primary headache disorders, their association with exacerbation of headache by pressure applied over paranasal sinuses could not be verified. However, there was a significant association between longer COVID-19 duration (non-headache symptoms persistence at second assessment) and pain exacerbation by pressure applied over the paranasal sinuses at the first visit (*p* = 0.003).

Nasal symptoms occurred in 40% (anterior or posterior discharge), 30% (congestion) and 42% (hyposmia/anosmia) of patients. These symptoms persisted until the second consultation in 33% of the group. Apart from rhinorrhoea and nasal congestion, other cranial autonomic symptoms (CAS) occurred in 15% of patients with headache. Clinical diagnosis of ARS according to EPOS criteria could be established in 45% of cases. ICHD-3 criteria allowed for headache to be attributed to ARS in 51% of cases. ARS was associated with headache (*p* = 0.003) (Table 3) and OR for headache in ARS group was 3.46 (CI = 1.47–8.14). In regard to pain intensity and quality, the ARS group was not significantly different from non-ARS subjects (*p* > 0.05). (Table 4) Fever and cough were reported respectively by 55 (42%) and 50 (38%) patients at first assessment, but neither of these symptoms showed significant association with headache (Table 5) or its phenotype (*p* = 0,54 in cough and *p* = 0,64 in fever).

**Table 3.** Comparison between patients with and without ARS.

	ARS present *n* = 58	ARS absent*n* = 72	*p*-value
Demographic characteristic			
Age (median)	39.50	39.00	0.89
Sex (female) *n* (%)	33 (57)	47 (65)	0.43
History of migraine/TTH *n* (%)	15 (26)/7 (12)	13 (18)/14 (19)	0.26
Clinical characteristic			
Headache phenotype (migraine/TTH) *n* (%)	15 (26)/33 (57)	12 (17)/30 (42)	0.96
Pain exacerbated by pressure applied over the paranasal sinuses *n* (%)	13 (22)	12 (17)	0.55
non-nasal CAS *n* (%)	8 (14)	7 (10)	0.66
Headache attributed to ARS according to ICHD-3 *n* (%)	47 (81)	0 (0)	<0.001**
cough *n* (%)	30 (52)	20 (28)	0.009**
fever *n* (%)	22 (38)	33 (46)	0.47

SD – standard deviation, ARS – acute rhinosinusitis, CAS – cranial autonomic symptoms, TTH – tension-type headache, ICHD-3 – 3rd edition of International Classification of Headache Disorders. *p-value </= 0.05. **p-value </= 0.01.

**Table 4.** Association between ARS and headache/facial pain.

	*p*-value	OR (95% CI)
Headache	0.003	3.46 (1.47–8.14)
Pain over paranasal sinuses	0.056	2.48 (1.06–5.80)
Exacerbation of headache by pressure applied over paranasal sinuses	0.547	1.44 (0.602–3.46)

OR – odds ratio, CI – confidence interval, ARS – acute rhinosinusitis.

**Table 5.** Comparison between patients with and without headache and/or facial pain.

	Headache and/or facial pain present*n* = 93	Headache and/or facial pain absent*n* = 37	*p*-value
Demographic characteristic			
Age (mean + SD)	40.40 + 14.87	43.08 + 18.42	0.43
Sex (female) *n* (%)	61 (66)	19 (51)	0.19
History of migraine/TTH *n* (%)	24 (26)/13 (14)	4 (11)/8 (22)	0.11
Clinical characteristic			
ARS *n* (%)	49 (52)	9 (24)	0.003**
Smell impairment *n* (%)	30 (32)	13 (35)	0.91
Pain exacerbated by pressure applied over the paranasal sinuses *n* (%)	25 (27)	0 (0)	0.001**
non-nasal CAS *n* (%)	13 (14)	2 (5)	0.28
cough *n* (%)	36 (39)	14 (38)	1.00
fever *n* (%)	35 (38)	20 (54)	0.13

SD – standard deviation, ARS – acute rhinosinusitis, CAS – cranial autonomic symptoms, TTH – tension-type headache. *p-value </= 0.05. **p-value </= 0.01.

## Discussion

### Pathogenesis of COVID-19-related headache

This study demonstrates that in line with ICHD-3, headache can be attributed to ARS in half of COVID-19 patients. Moreover, odds for headache are 3.5 times higher in subjects with sinonasal inflammation during SARS-CoV-2 infection. Since smell impairment is part of ARS characteristics that might explain why previous research showed an association between anosmia and headache (9,10). However, it must be stressed that headache cannot be attributed to ARS in the remaining half of COVID-19 cases. Does that mean that there exist at least two different pathways for COVID-19 related headache? If that were the case, it could be expected that these two pathways led to a headache of at least partially different phenotype. To check this hypothesis, we compared the phenotype of COVID-19 related headache with and without ARS. However, we found no statistically significant differences other than nasal symptoms. This would suggest a common mechanism for headache in COVID-19 regardless of sinonasal involvement. Consequently, it seems that COVID-19 headache is not a symptom of ARS, but rather a systemic component of this viral infection. This observation would go along with the current understanding of ARS, which overlaps with disorders not restricted to the nose and paranasal sinuses (e.g. common cold, influenza). Interestingly, headache in COVID-19 seems not to be directly related to systemic infection severity, since an association between fever and headache was not found in this study. In this regard our study reflects the results obtained by other researchers (4).

However, there remains the question why nasal symptoms are much more prevalent in headache vs. headache-free COVID-19 subjects. Several explanations for this observation are possible. Firstly, nasal congestion and rhinorrhoea (discharge) could be in fact CAS, due to trigeminal autonomic reflex activation by central or meningeal mechanisms. To verify this hypothesis, we have assessed non-nasal CAS presence. However, these other CAS had low prevalence and were not associated with headache in COVID-19. Apart from CAS, we also considered a shared mechanism behind headache and ARS in COVID-19. In this hypothesis, both nasal symptoms and pain would occur only if COVID-19 had a certain level of severity. In this situation subjects without ARS and headache would have different disease features. This association was however not found in our analysis, although that could be due to small size of a subgroup without headache and ARS (*n* = 9). This rationale may indicate that headache in COVID-19 is caused by a systemic viral infection and facilitated by sinonasal mucosa inflammation. In fact, the theory of rhinosinusitis causing COVID-19-related headache seems to be compelling also to other authors. Some of them have proposed a theory on the underlying mechanism of this phenomenon (4,9). In this hypothesis, rhinosinusitis leads to the activation of the trigeminovascular system, and henceforth the activation of nociceptive pathways in the brain, leading to a series of events similar to migraine. Recently this argument has received support from research on an animal model. In this study, the inflammatory stimulus (bradykinin) applied close to the nasal mucosa led to activation of the spinal trigeminal nucleus (11). Furthermore, it should be mentioned that this concept would also explain why medications addressing nasal mucosa are effective in reducing headache in ARS (i.e. intranasal corticosteroids (12), decongestants (13)). Finally, it should be underlined that our study does not confirm causality between ARS and headache despite the above described rationale.

### Classification of COVID-19-related headache

Assuming that headache in COVID-19 results from a systemic viral infection requires confirmation by ICHD-3 criteria. Our study demonstrates that the majority of subjects with COVID-19 fulfil these criteria, although the exclusion of meningitic or encephalitic involvement was not confirmed by imaging or cerebrospinal fluid tests. Indeed, the majority of our patients (89%) observed the onset of headache with other COVID-19 symptoms (C.1). Moreover, these symptoms subsided alongside headache in 96% of cases (C.3). These results correspond with a recently published hospital-based study (14,15). In that research, the criterium C.1 was met by 74% of patients, although only 55% of subjects met criterium C.3. The latter observation may be explained by more severe disease course in that study (hospitalised subjects). Other similarities to this study include moderate to severe intensity of pain (C.4.a) experienced by the majority of patients (87% in our research). Also in accordance with Trigo López et al. (14), forehead pain location (59%) was more prevalent in our cohort than diffuse pain required by ICHD-3 criteria (C.4.b).

It remains unresolved whether headache in COVID-19 could also be related to direct viral damage of the central or peripheral nervous system during infection. On one hand, there is a growing number of reports on meningitis, encephalitis or encephalopathy during COVID-19 (16,17). Futheremore, studies in vivo described a mechanism of virus infiltration of central nervous system (18). On the other hand, a large case series found few such cases among 4,491 neurological patients (19). Also studies analysing cerebrospinal fluid samples or post-mortem results in COVID-19 subjects indicate that virus presence in the central nervous system is observed only to a limited degree (20,21). Therefore, more robust evidence correlating virus infiltration with pain is required to support this mechanism in COVID-19-related headache.

As mentioned above, rhinosinusitis is part of COVID-19 symptoms in half of the patients. Consequently, headache in COVID-19 can be often attributed to ARS. ICHD-3 criteria allow for such attribution through parallel onset and remission of headache and ARS symptoms. However, our research uncovered that ICHD-3 C.3 and C.4 criteria have little sensitivity in COVID-19-related ARS (6).

In regard to C.3, only 28% of ARS patients showed exacerbation of headache by pressure applied over the paranasal sinuses (6). The statistical analysis proves no significant association (*p* = 0.55), which indicates low sensitivity of this symptom. What is more, the specificity of headache exacerbation by touch in viral ARS is unproven, especially in the light of a study where ARS subjects had hypersensitivity to touch applied over paranasal sinuses comparable to chronic rhinosinustitis and chronic fatigue syndrome (22). Moreover, such hypersensitivity was present in 70% of subjects misdiagnosed with ‘sinus headache’ in another study (23). The latter patients were finally diagnosed with migraine or TTH. Admittedly, ‘hypersensitivity to touch’ differs from ‘exacerbation of headache in response to pressure’. However, the practical value of this symptom seems limited, especially when myofascial tenderness in TTH and allodynia in migraine are taken into account. Therefore, it seems that the role of criterium C.3 should be limited to bacterial ARS where facial pain may point to maxillary sinus inflammation (24). However, bacterial infection is responsible for only 0,5–2% of all ARS cases (7), which further undermines the value of C.3 criterium in the whole ARS spectrum. Finally, our study found that exacerbation of headache by pressure applied over paranasal sinuses was associated with longer COVID-19 duration (*p* = 0.003). The reason for this phenomenon is unclear, especially since other disease characteristics at first visit were not related do persistent symptoms.

In classification of ARS-related headache, criterium C.4 states that headache should be ipsilateral to unilateral rhinosinusitis. In our study we have not registered any cases of this symptom. Although unilateral headache was observed in 7% of cases, other nasal symptoms were bilateral in these subjects. Hence the C.4 condition was not met. In addition to this observation, rhinological studies again support the value of this symptom only in rare bacterial ARS of maxillary sinus (24). Moreover, several other studies found no association between sinusitis location on computed tomography and perceived pain location (25–27). Lastly, unilateral, often localised pain is considered a red flag in ARS, as it may indicate serious extranasal complications (7). In this regard application of ICHD-3 criteria could induce false belief in a simple case of ARS when serious pathology diagnosis is postponed.

Despite the above described controversies, it must be stressed that the data provided by our study cannot be extrapolated to all ARS cases. Future research should validate ICHD-3 criteria for ARS in infections caused by viruses other than SARS-CoV-2. This research should also look for alternative criteria of higher specificity and sensitivity. Our study suggests that osmophobia may be an optimal candidate for such a symptom. On the one hand, it had very low prevalence in COVID-19-related ARS. On the other hand, it has a high prevalence in migraine (28). Moreover, osmophobia is almost the opposite to smell impairment observed in both acute and chronic rhinosinusitis, therefore it would help to distinguish apparent rhinosinustitis from its notorious true cause – migraine (29). Other authors propose also a phenotype driven approach to secondary headache classification (30). However, our results do not support specific ARS related headache phenotype when compared with non-ARS COVID-19 subjects.

On a side note, it should be said that rare instances of purely facial COVID-19-related pain in our study have been left unclassified. The reason for that is that the 1st edition of International Classification of Orofacial Pain (ICOP) has no chapter devoted to systemic viral infection nor rhinosinusitis (31). This situation will be hopefully amended in the future editions of this important document.

### Comparison with other studies

Other studies in similar cohorts of mostly mild and moderate COVID-19 cases showed prevalence of headache in 66–82% subjects, nasal congestion in 34–68%, nasal discharge in 51–60% and hyposmia/anosmia in 55–70% (1,3,32). In our study these numbers were similar for headache (72%), but considerably lower for other nasal symptoms: 30% for congestion, 40% for discharge and 42% for smell impairment. It is probably a consequence of methods applied in our study, where only symptoms present during the consultation were recorded. Incidentally, a detailed symptom timeline published by O’Keefe at al. (3) shows that the prevalence of nasal congestion was 43% and loss of smell 37% on the third day from disease onset. These results are comparable to our cohort, where the first assessment was performed at third day (median). Moreover, some patients reported losing and regaining the sense of smell in the period between assessments, which was not taken into account in our study. This observation can be supported by findings from Lechien et al. (1), who noted that the duration of anosmia/hyposmia lasted 8.41 ± 5.05 days. Since in our study the second assessment was performed at mean 9 days after the first consultation, that could be the case in some of the subjects. Another explanation for differences in the prevalence of nasal symptoms could be associated with new variants of SARS-CoV-2, although no scientific data published so far confirms different symptoms of these virus types. Nevertheless, it is noteworthy that according to the Polish Medical Research Agency, especially type 20I/501Y.V1 (B.1.1.7) was highly prevalent in our catchment areas during the recruitment period. The prevalence of SARS-CoV-2 variants differed also between regions where our practices are located (distance of 500 km). That could explain the difference in ARS prevalence found between the investigators.

In our study TTH phenotype was observed in 68% of subjects, and migraine phenotype in 29%, which was similar to the results from the Spanish group (14). Pre-existing primary headache was not associated with COVID-19-related headache phenotype in our study. This observation was reflected by results from two other groups (15,33). However, this seems to stand in opposition to the reports of migraine exacerbations after COVID-19 infection. In fact, our group also published a description of a case series where migraine exacerbation after SARS-CoV-2 infection was observed (34). This discrepancy warrants further research analysing subjects from different cohorts or disease course.

## Limitations

This study describes COVID-19 symptoms in patients recruited in large proportion from general practice. This means that subjects probably reflected the general population to a larger extent than in hospital-based studies. However, this also means that similar to the general population, the majority of cases were mild. Moreover, it should be underlined that progression to severe COVID-19 occurs usually in the second and third week of disease. This means that some of the cases that required hospitalisation were not assessed within the mean 13-day timeframe of our study. As a result, the group of severe COVID-19 cases was too small to allow for an analysis of associations between headache or ARS and hospitalisation rate, hypoxemia or mortality.

Recruiting some patients from neurologic consultations was another limitation of this study. On the one hand, this allowed us to cover both entry points into Polish healthcare system for subjects with early COVID-19 symptoms (primary and hospital-based care). On the other hand, this might have resulted in the inclusion only of subjects referred for neurologic consultation in the hospital-based part of our study. To discount this bias risk, a neurologist (MWP) included all the consecutive COVID-19 patients that approached the emergency or outpatient ward within investigator duty hours. We found only one significant difference between the primary and specialist care recruited groups – ARS was more prevalent in general practice.

Regardless of the above mentioned steps undertaken for lowering recruitment bias risk, it must be stressed that this study had other limitations in the inclusion process. Firstly, only subjects seeking medical help from participating investigators were included. In other words, patients could not be recruited if for any reason they refrained from medical consultation. To our knowledge, literature does not provide a profile of such patients. However, it seems justified to assume that this group would include asymptomatic cases, a higher percentage of subjects with very mild symptoms or members of society sceptic of the COVID-19 pandemic. Not included in the study were also proportionately small groups of patients in specialised healthcare (e.g. nursing homes, hospices), as well as subjects with very severe disease admitted directly to intensive care units and diagnosed then. The latter two groups consist of patients at the highest risks of severe COVID-19. Therefore, their exclusion could to some extent explain low hospitalisation rate and no mortality in our study group.

Our study was limited by the lack of nasal endoscopic examination or imaging of paranasal sinuses. However, several factors support this approach. Firstly, the clinical diagnosis of ARS based solely on symptoms has high sensitivity according to EPOS (7). Its low specificity is in this case discounted by the positive results of SARS-CoV-2 genetic or antigen tests – confirmed COVID-19 minimises the probability of other acute rhinological disorders. Secondly, performing computed tomography and nasal endoscopy in all subjects was considered unethical due to radiation exposure and increased epidemic risk associated with these procedures in homebound patients. Moreover, this study is limited by the fact that significant number of assessments took the form of teleconsultations. While this probably did not influence the results of interviews, it might have limited the value of facial palpation assessment. However, we found that the type of consultation was not associated with prevalence of headache exacerbation by palpation over paranasal sinuses. Control of medications taken by patients may be regarded as a limitation of this study. On one hand, at patient inclusion many disorders affecting neurologic and rhinologic status were excluded, as well as prior use of corticosteroids. Also, all subjects were only prescribed medications according to Polish guidelines valid at the time of trial (i.e. no corticosteroids). However, the predominantly ambulatory setting of this study made it difficult to prevent supplementation of other medications taken without physicians’ knowledge.

Finally, the results of this study reflect findings in early, mostly mild COVID-19 cases treated predominantly in outpatient setting. Above mentioned similarities to hospital-based studies indicate that these results could be also generalised to more severe cases. However, it must be underlined that the COVID-19 pandemic is evolving with new SARS-CoV-2 variants emerging that could differ in clinical presentation. Also subgroups of patients not included in our study (very severe cases, hospices, very mild symptoms) could have different disease presentation. Even more importantly these results cannot be generalised to ARS cases of other aetiologies (e.g. rhinoviruses, other types of coronaviruses).

## Conclusions

Headache in COVID-19 is a highly prevalent symptom. There is also a strong association between rhinosinusitis and headache in SARS-CoV-2 infection, although the pain mechanism lies probably in a systemic reaction to virus. Therefore in some cases COVID-19-related headache occurs without ARS. This implicates that rhinosinusitis is a component of COVID-19-related headache, but not a mandatory one.

This study also indicates the need to adjust ICHD-3 criteria for headache attributed to ARS to the current understanding of the acute sinonasal infection. Our data, as well as previously published research, suggest low diagnostic value of ‘exacerbation of headache by pressure applied over paranasal sinuses’ and ‘ipsilaterality of pain and sinusitis’. Future studies should try to validate these symptoms in infections other than COVID-19. This research should look for new, more sensitive and specific symptoms that would help distinguish between ARS and primary headache. Osmophobia is a good candidate in this regard. There is also a need for updating ICOP criteria to allow for the classification of COVID-19 and ARS related facial pain.

## Clinical implications

- Rhinosinusitis is associated with headache in COVID-19
- COVID-19 may cause strictly facial pain in rare cases
- There is a need for more precise criteria of headache and facial pain attributed to ARS
